# Contrasting Attitudes towards Animal Welfare Issues within the Food Chain

**DOI:** 10.3390/ani3020551

**Published:** 2013-06-10

**Authors:** Fabio Napolitano, Maria Serrapica, Ada Braghieri

**Affiliations:** Scuola di Scienze Agrarie, Forestali, Alimentari ed Ambientali, Università degli Studi della Basilicata, Via dell’Ateneo Lucano 10, 85100 Potenza, Italy; E-Mails: maria.serrapica@unibas.it (M.S.); ada.braghieri@unibas.it (A.B.)

**Keywords:** animal welfare, citizen attitude, consumer behavior, animal-based products

## Abstract

**Simple Summary:**

Intensive systems have been increasingly considered to be responsible for a dramatic reduction in animal welfare. As a consequence, large segments of animal welfare-sensitive consumers have been identified. On the other hand, price conscious consumers, if accepting higher prices, are more likely to require explicit justification of returns in quality. Therefore, scientifically validated monitoring systems for assessing the welfare of farm animals have been developed in order to provide a certification system, allow the differentiation of animal-based products through constant and reliable signaling systems, and promote animal welfare friendly farming systems.

**Abstract:**

Intensive systems have facilitated the production of animal-based products at relatively low prices. On one hand, these methods have been increasingly considered to be responsible for a dramatic reduction in animal welfare, as indicated by the high prevalence of stereotypies in sows, brittle bones in hens, lameness in broilers and short life span in dairy cattle. As a consequence, large segments of animal welfare-sensitive consumers have been identified. On the other hand, price conscious consumers, if accepting higher prices, are more likely to require explicit justification of returns in quality. Therefore, scientifically validated monitoring systems for assessing the welfare of farm animals have been developed in order to provide a certification system, allow the differentiation of animal-based products through constant and reliable signaling systems, and promote animal welfare friendly farming systems.

## 1. Contrasting Definitions of Animal Welfare

Intensive animal rearing systems have allowed the production of animal-based products at relatively low prices, facilitated by cheap feed grains, limited grazing land, readily available antibiotics and increased production efficiency. As a consequence, over the last 60 years, human consumption of meat, milk, and eggs has risen both in industrial countries and in the developing world. Globalized trade and media, lower animal product prices and urbanization have helped make animal protein-rich diets more common.

The intensification of farming practices contributed to the formation of different opinions about animal welfare and the way it should be assessed. A common approach to animal welfare is based on the biological functioning of animals in terms of productivity, growth and health. This view does not take into account the behavioral needs and feelings of the animals. Therefore, although not natural and restraining, any farming technique can be considered acceptable as long as animals remain productive (e.g., sow stalls are causative of stereotypies but they can be still deemed adequate because of the positive effects on productive efficiency; a very short life-span in dairy cattle can be considered an acceptable consequence of high production levels; *etc*.). Other scientists, however, rely on the affective states of the animals and on the ability to express their natural behavior in order to define animal welfare [1]. Thus, based on different assumptions, scientists can achieve contrasting results in terms of acceptability of farming practices [2]. These contrasting results are likely to be reflected in contrasting consumer attitudes.

## 2. Reasons for High Sensitivity to Animal Welfare Issues

The intensive agricultural methods have been increasingly considered to be responsible for some public health issues (e.g., BSE), various ecological problems concerning air and water pollution, loss of livestock genetic diversity and conversion of animals into short-lived production machines [3], currently referred to as ‘hidden costs’ [4]. As to animal welfare in particular, different stakeholders have contributed to its deterioration (e.g., lobby-sensitive governments, production efficiency oriented science and consumerism), including retailers [5]. The latter have imposed pressure on farms to increase production efficiency and cut production costs, which has resulted in higher retailer profits, as farms have little control over margins [5]. In fact, according to the Consumer Price Index, retail costs to consumers for meat and dairy products in the United States have increased by approximately 45% since 1982–1984, but payments to farmers have not increased at all [6]. In some cases they have decreased; for example, farmers in United States received an average of $74.60 for 100 lbs of cattle marketed in 1990 but only $58.70 in 1996.

One of the latest enterprises moving towards industrialization has been agriculture. Since the first animal domestications, when humans changed from migrant hunter-gatherers to settled farmers, animal production techniques were basically unchanged until the 1940s, as they were mainly based on the extensive use of pasture. Subsequently, several changes occurred in animal housing and handling, along with the adoption of performance enhancing techniques, due to advances in nutrition, veterinary care and genetic selection. Crates for sows, battery cages for hens, selective breeding for rapid growth in broilers, and milk production in dairy cattle are some examples of new rearing techniques determining increased production efficiency. However, they are also responsible for a dramatic reduction of animal welfare, as indicated by the corresponding outbreaks of stereotypies in sows, brittle bones in hens, lameness in broilers and short life span in dairy cattle [7]. Factory farm methods of raising and slaughtering animals represent the main production system in Europe and North America, and are also acquiring most of the market in developing countries.

There is thus no question that industrialized agriculture is responsible for markedly changed farming systems, which in turn has led to increased sensitivity to animal welfare issues. In a study performed by the European Commission [8], citizens were asked to rate the importance of farm animal protection on a scale ranging from 0 to 10. The majority of citizens answered towards the high end of the scale with a mean score of 7.8. At a legislative level, in response to this public endorsement, an increasing number of regulations on the welfare of farm animals have been issued in the European Union. In North America, livestock welfare has traditionally been an area for industry self-regulation. However, a number of opinion polls have revealed public interest in the way that farm animals are raised [9].

The growing public interest in the way animals are raised is related, along with the marked reduction of animal welfare induced by intensive farming, to several other factors, such as wealth of western countries and dissociation of modern societies from the agricultural context. In addition, for most of the past century, western societies have often directed their ethical attention on oppressed or ignored humans. More recently, the achievement of several goals in these fields has meant that the focus has been turned on the treatment of the non-human world, including farm animals [10]. A large segment of animal welfare-sensitive consumers (55%) has been identified by Vanhonacker *et al.* [11]. Expectations induced by the information on animal welfare were able to affect the quality perception of lamb meat [12] and beef [13]. In addition, information about animal well-being was a major determinant of consumer willingness to pay yogurt [14]. However, to be effective, this information should be paired with products presenting a good eating quality because, along with information, sensory properties play an important role in affecting consumer willingness to buy animal-based food products.

## 3. Reasons for Low Sensitivity to Animal Welfare Issues

According to a paper published in Nature in 2005 [15], science suffers from an asymmetrical approach when the animal kingdom is studied: “scientists have had no problem seeing… experiments using mice held dangling from their tails as models for human stress… despite the tailless condition of *Homo sapiens*. But when it comes to applying conclusions about the human psyche to that of an animal, science draws a well-worn line”, although “continuity across species at a structural level is unquestioned in biomedical research”. This attitude is widely applied to farm animals and when productivity issues are tackled little consideration is given to animal welfare per se, as no value is attributed to it that is not already expressed in the value of what is produced [16].

At a market level, a similar contradiction has been initially described by Grunert [17] in individuals behaving differently as either consumers or citizens when it comes to animal-based products. As citizens, they tend to approach socially sensitive topics, such as animal welfare, in a way that conforms to perceived social norms (*i.e*., showing high sensitivity to animal welfare issues), whereas as consumers, individuals will often buy products obtained from systems criticized about their welfare standards, even when alternative products are available [18]. In addition, most studies assessing consumer willingness to pay for high welfare animal-based products are based on direct questioning, which is deemed responsible for a social desirability bias, whereas indirect questioning (e.g., consumers are asked to indicate what they believed the average citizen would choose) may help in obtaining more accurate representations of people’s opinions [19] by reducing social influence. The rationale behind this is that people are less concerned with answering in ways that make other people look better [20].

Price-conscious consumerism may be another reason for individuals not buying animal welfare-friendly products. In fact, from a strict economic point of view, the values of animal welfare are negative, inducing unmitigated costs to producers and consumers [21]. Results from correlation studies support the hypothesis of an inverse relationship between price consciousness and product involvement, and the hypotheses that price consciousness and product involvement have opposite implications for several price-related constructs [22]. However, the relationship between animal welfare and production costs is complex [23]. For instance, it has been estimated that only 19% of the price paid by consumers reaches farmers [24]. According to Appleby [23] and McInerney [16], marked improvements in farm animal welfare could be achieved with only minor increments of food prices (less than 1%), whereas Bornett *et al*. [25] noted that moving from fully slatted floors to Freedom Food standards for pigs determines a 4% increment in pork production costs.

In addition, animal welfare, as with other ethical aspects, is a credence characteristic that cannot be confirmed either before or after purchase [26]. In cases where price conscious consumers do accept higher prices, they are more likely to require explicit justification of returns in quality and are less likely to be simply operating on an inference-based ‘higher price means higher quality’ scheme. 

A system of certification of higher quality in terms of ethical issues related to animal welfare as well as to environmental concerns, sustainable production, livestock biodiversity and product safety, may be considered the organic farming labeling according to European Council Regulations 834/2007 [27] and 889/2008 [28]. Various sources show dynamic increase of organic food consumption in EU Member States, particularly in France, Germany, Italy and the United Kingdom, which are the four largest markets in the EU, indicating a growing consumers’ confidence toward this production system and certification even in a food market at the moment largely dominated by uncertainty. 

## 4. Future Trends

At a productive level, several food companies label their products, suggesting that the animals used for that purpose were kept in welfare-friendly farming conditions, thus generating unfair competition. As producers cannot provide verification of this credence attribute (animal welfare) through traditional testing methods, a number of codes of practice and farm assurance schemes (e.g., Freedom Food in the UK) have been issued focusing on the welfare of farm animals reared for food in the attempt to correct this informational asymmetry.

However, these are only standards and, as such, are not able to verify the real welfare state at animal level. More recently, within the Welfare Quality^®^ project which involved several European and non-European countries, three different scientifically validated monitoring systems for assessing the welfare of cattle, pigs and poultry [29,30,31] at farm level have been developed to provide a certification system. However, only if the underlying organizations succeed in establishing a quality reputation in markets will the corresponding labels be accepted as a quality surrogate. 

In western countries, animal production enterprises are facing a progressive saturation of markets which are consequently becoming very competitive. Product differentiation can be based on either product or process characteristics. For animal-based foods, process characteristics are represented by farming practices and related animal welfare conditions, which, therefore, possess great potential for differentiation through constant and reliable signaling systems and appropriate information given to consumers [32].
